# Glioma cells showing *IDH1* mutation cannot be propagated in standard cell culture conditions

**DOI:** 10.1038/bjc.2011.27

**Published:** 2011-02-15

**Authors:** S Piaskowski, M Bienkowski, E Stoczynska-Fidelus, R Stawski, M Sieruta, M Szybka, W Papierz, M Wolanczyk, D J Jaskolski, P P Liberski, P Rieske

**Affiliations:** 1 Department of Molecular Pathology and Neuropathology, Chair of Oncology, Medical University of Lodz, Czechoslowacka 8/10 Street, Lodz 92-216, Poland; 2 Department of Pathomorfology, Medical University of Lodz, Czechoslowacka 8/10 Street, Lodz 92-216, Poland; 3 Department of Neurosurgery, Medical University of Lodz, Kopcinskiego 22 Street, Lodz 90-153, Poland

**Keywords:** *IDH1*, astrocytic tumours, glioblastoma, cell line, *EGFR*, R132H

## Abstract

**Background:**

It has recently been reported by several sources that original (i.e., present *in vivo*) glioma cell phenotypes or genotypes cannot be maintained *in vitro*. For example, glioblastoma cell lines presenting *EGFR* amplification cannot be established.

**Methods and results:**

*IDH1* sequencing and loss of heterozygosity analysis was performed for 15 surgery samples of astrocytoma and early and late passages of cells derived from those and for 11 archival samples. We were not able to culture tumour cells presenting *IDH1* mutations originating from currently proceeded 10 tumours; the same results were observed in 7 samples of archival material.

**Conclusion:**

The *IDH1* mutation is expected to be almost mutually exclusive with *EGFR* amplification, so glioma cells with *IDH1* mutations seem to represent a new group of tumour cells, which cannot be readily analysed *in vitro* because of their elimination. The reasons for this intriguing phenomenon should be investigated since its understanding can help to define a new therapeutic approach based on simulating *in vivo* conditions, responsible for tumour cells elimination *in vitro*. Moreover, a new model for culturing glioma cells *in vitro* should be designed since the current one does not provide conditions corresponding to *in vivo* growth.

The discovery of *IDH1* mutations was very significant for neuro-oncologists. So far, mutations of *IDH1* have been detected almost exclusively in glial tumours (Bleeker *et al*, 2009), including astrocytomas and secondary glioblastomas, but not in primary glioblastomas (Watanabe *et al*, 2009). In addition, the function of *IDH1* does not seem to be typical for a tumour suppressor, a classical oncogene, or a mutator. The IDH1 protein is required for NADPH production and lipid synthesis (Kloosterhof *et al*, 2011). All of these factors have attracted a lot of attention to *IDH1* analyses, including *in vitro* investigations. Nevertheless, it appears difficult to propagate cells showing the *IDH1* mutation *in vitro* since they are easily eliminated under standard cell culture conditions.

## Materials and methods

### Tumour samples

The study included 26 (15 cultured and 11 archival) cases of astrocytic tumours (17 – diffuse astrocytoma, 9 – anaplastic astrocytoma) diagnosed at the Department of Pathomorphology, Medical University of Lodz according to the World Health Organisation criteria for classification of brain tumours. Tissue samples were obtained from patients with astrocytic tumours treated in the Department of Neurosurgery, Medical University of Lodz, Poland.

### Cell cultures/DNA isolation

Fifteen specimens were cultured. The tumour cells were dispersed by means of collagenase type IV (200 U ml^–1^, 37 °C for 6 h). Subsequently, the cells were cultured in alpha MEM media containing 10% FBS. After 48 and 96 h, the medium was changed and part of the cells was isolated for DNA analysis. DNA was isolated from snap-frozen tissues (stored at –80 °C) and from cells harvested after the first and the second passage. On the first and the second day, DNA samples were also isolated from cells floating (dead) in the culture medium for the purpose of the DNA profile analysis. The first harvesting was performed no longer than 2 days after cells culturing. For loss of heterozygosity (LOH) analysis, DNA was isolated from blood samples. All DNA samples were extracted by means of a Macherey–Nagel DNA purification kit (Macherey-Nagel, Düren, Germany). All types of cultured cells, including floating cells, were tested for Trypan blue absorbing.

### Archival sample analysis

Eleven samples of astrocytoma with visible karyotypic changes (collected in 1990s) were verified for *IDH1* mutation presence. DNA was extracted from snap-frozen tumour tissues (stored at −80 °C) and from microscopic specimens derived for karyotyping from cell cultures after the second passage. All DNA samples were extracted by means of a Macherey–Nagel DNA purification kit.

### *IDH1* sequencing

Exon 6, including codon 132 of the *IDH1* gene, was amplified by PCR and sequenced using the dideoxy termination method and SequiTherm Excel DNA Sequencing Kit (Epicentre Technologies, Madison, WI, USA). The primers used for PCR amplification of the DNA sequences were: *IDH1* – 5′-GGCACCCATCTTCTGTGTTT-3′ (sense) and 5′-ATATATGCATTTCTCAATTTCA-3′ (antisense). The sequencing primers used were: *IDH1* exon 6 – 5′-GCAAAAATATCCCCCGGCTT-3′ (sense), and *IDH1* exon 6 – 5′-CGGTCTTCAGAGAAGCCATT-3′ (antisense). A Li-Cor automatic sequencer system (Li-Cor, Lincoln, NE, USA) was applied for the separation and analysis of PCR-sequencing products.

### LOH analysis: microsatellite analysis to classify type of *in vitro* surviving cells (normal *vs* tumour)

The specimens were examined for LOH using pairs of tumour specimens and corresponding peripheral blood samples. The markers were selected using the NCBI database (D1S508, D1S199, D1S197, D1S162, D1S2734, D1S2720, D1S429, D1S510, D10S1709, D10S209, D10S587, D10S197, D10S1267, D10S587, D18S481, D22S303, D22S257, D22S1163 and D22S1150) and obtained from MWG-Biotech AG (Ebersberg, Germany). PCR products were visualised with a Li-Cor automatic sequencer. Loss of heterozygosity was judged to be present if the allelic signal intensity of the tumour sample was reduced by at least 50% relative to the corresponding allele in the patient's control DNA.

## Results

### *IDH1* mutations in frozen tumour cells and corresponding cell cultures

#### Cell cultures

**Figure 1:**
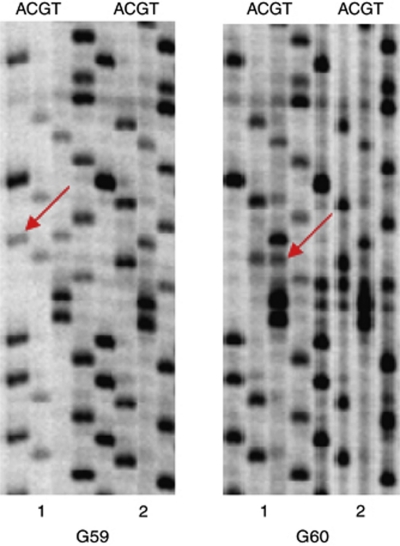
*IDH1* gene sequencing analysis of two astrocytic tumours (G59 and G60). The analysis of DNA from frozen samples shows a heterozygous mutation; both mutated and wild-type nucleotides are detected (1). The analysis of the second passage of corresponding culture cells shows the lack (G59), or almost invisible band (G60) of the mutated nucleotide (2). Mutated nucleotides are marked with arrows.

DNA sequencing revealed *IDH1* mutations in 10 surgical specimens of astrocytic tumours coming from 15 patients. The mutated template was no longer detectable in adherent cells grown *in vitro* (in four cases after the first passage and in six cases after the second passage) derived from the tumour samples with *IDH1* mutations (Figure 1). Free floating cells (absorbing Trypan blue) from the medium showed *IDH1* mutation after the first and the second passage. Our previous investigations showed that none of the tumours presented *EGFR* amplification, and three showed a mutation of *TP53*.

In eight cases, cell cultures derived from specimens originally presenting the *IDH1* mutation survived at least 10 passages but were never stabilised. In five of those cases, LOH was observed in the initial culture. Only in one case, tumour cells presenting LOH were observed after disappearance of cells presenting *IDH1* mutation. It suggested that after elimination of cells with *IDH1* mutation only normal cells remained in the majority of the cell cultures. Two out of five astrocytomas presenting lack of *IDH1* mutation (after the analysis of surgery sample and early cell culture sample), showed LOH of several markers. Cells coming from one of them cultured in standard cell culture conditions still show LOH of 1p (after 20 passages – possible stabilisation). In the second case, we did not detect in the standard cell culture conditions LOH observed *in vivo.*

#### Archival sample analysis

In all, 7 out of 11 DNA samples isolated from frozen tumour tissues showed *IDH1* mutations in the sequencing analysis. The quality and amount of archival DNA was not sufficient to perform other molecular analyses such as LOH, and so on. Nonetheless, in six corresponding DNA samples isolated from cultured cells in 1990s after the second passage, *IDH1* mutation was not visible. In one remaining case, mutated bands were significantly weaker (almost invisible) than in the frozen tissue samples.

## Discussion

*IDH1* mutations seem to have a significant role in glial tumours (Ducray *et al*, 2009; Hartmann *et al*, 2009). Zhao *et al* (2009) showed that IDH1 regulates HIF1 expression. Recently, Sanson *et al* (2009) suggested that the *IDH1* mutation might correlate with a glial tumour grade. However, the specific role of the *IDH1* mutation in glial tumours, such as astrocytic tumours and secondary glioblastomas is unknown.

In this paper, we described obstacles in culturing glioma cells with *IDH1* mutations. What needs to be added, similar problems were described for *EGFR* amplification analysis in glioblastomas, that is, disappearance of *EGFR* amplification was observed in standard cell culture conditions. Elimination of *EGFR* amplification during glioblastoma cells culturing is a well-known phenomenon the causes of which, however, remain enigmatic (Pandita *et al*, 2004; Witusik-Perkowska *et al*, 2010). We observed two cases of astrocytoma without *IDH1* mutation presenting LOH for several markers in surgery samples (*in vivo*). *In vitro* proliferating (surviving) cells of one of them showed lack of LOH (putative normal) whereas cells of the second astrocytoma presenting LOH survived in standard cell culture conditions for 20 passages so far. In addition, glioma cell culture collections can be analysed, and none of the 15 commercially available cell lines presents *IDH1* mutation (Bleeker *et al*, 2009; Kloosterhof *et al*, 2011).

All collected data suggest that glioma tumour cells with *IDH1* mutation cannot be propagated *in vitro* in conditions suitable for survival of normal human cells or glioma cells without *IDH1* mutation. The *IDH1* mutation is expected to be almost mutually exclusive with *EGFR* amplification, so glioma cells with *IDH1* mutation seem to represent a new group of tumour cells, which cannot be readily analysed *in vitro* because of their elimination. Nonetheless, we do not suggest that *IDH1* mutation is responsible for cells *in vitro* elimination. Apparently, the presence of a *TP53* mutation does not protect glioma cells with *IDH1* mutation against factors responsible for cells elimination. Although we did not define that beyond any doubt, the cells that survived after the elimination of tumour cells with *IDH1* mutation are most likely normal cells. Surprisingly, the kind of cells surviving *in vitro* in case of gliomas presenting EGFR amplification has not been defined in spite of years of investigation. We did not detect genetic alterations in cells cultured after the elimination of cells showing *IDH1* mutation, except for one case. Moreover, *IDH1* mutation is probably the earliest stage of glioma tumourigenesis (Kloosterhof *et al*, 2011).

Defining cell culture conditions that would sustain the original phenotype and genotype of glioma cells is critical. The cell culture conditions currently used are artificial and thus unsuitable for the growth and analysis of glioma cells – causing numerous artefacts. Nonetheless, as normal cells seem to be more congruous to the standard cell culture conditions than glioma cells with *IDH1* mutation, the factors responsible for the elimination of glioma cells *in vitro* should be identified for possible pharmacological use.

## References

[bib1] Bleeker FE, Lamba S, Leenstra S, Troost D, Hulsebos T, Vandertop WP, Frattini M, Molinari F, Knowles M, Cerrato A, Rodolfo M, Scarpa A, Felicioni L, Buttitta F, Malatesta S, Marchetti A, Bardelli A (2009) IDH1 mutations at residue p.R132 (IDH1(R132)) occur frequently in high-grade gliomas but not in other solid tumours. Hum Mutat 30: 7–11 19117336 10.1002/humu.20937

[bib2] Ducray F, Marie Y, Sanson M (2009) IDH1 and IDH2 mutations in gliomas. N Engl J Med 360: 2248–2249 19469031

[bib3] Hartmann C, Meyer J, Balss J, Capper D, Mueller W, Christians A, Felsberg J, Wolter M, Mawrin C, Wick W, Weller M, Herold-Mende C, Unterberg A, Jeuken JW, Wesseling P, Reifenberger G, von Deimling A (2009) Type and frequency of IDH1 and IDH2 mutations are related to astrocytic and oligodendroglial differentiation and age: a study of 1010 diffuse gliomas. Acta Neuropathol 118: 469–474 19554337 10.1007/s00401-009-0561-9

[bib4] Kloosterhof NK, Bralten LB, Dubbink HJ, French PJ, van den Bent MJ (2011) Isocitrate dehydrogenase-1 mutations: a fundamentally new understanding of diffuse glioma? Lancet Oncol 12: 83–91 20615753 10.1016/S1470-2045(10)70053-X

[bib5] Pandita A, Aldape KD, Zadeh G, Guha A, James CD (2004) Contrasting *in vivo* and *in vitro* fates of glioblastoma cell subpopulations with amplified EGFR. Genes Chromosomes Cancer 39: 29–36 14603439 10.1002/gcc.10300

[bib6] Sanson M, Marie Y, Paris S, Idbaih A, Laffaire J, Ducray F, Hallani SE, Boisselier B, Mokhtari K, Hoang-Xuan K, Delattre JY (2009) Isocitrate dehydrogenase 1 codon 132 mutation is an important prognostic biomarker in gliomas. J Clin Oncol 27: 4150–4154 19636000 10.1200/JCO.2009.21.9832

[bib7] Watanabe T, Nobusawa S, Kleihues P, Ohgaki H (2009) IDH1 mutations are early events in the development of astrocytomas and oligodendrogliomas. Am J Pathol 174: 1149–1153 19246647 10.2353/ajpath.2009.080958 PMC2671348

[bib8] Witusik-Perkowska M, Rieske P, Hulas-Bigoszewska K, Zakrzewska M, Stawski R, Kulczycka-Wojdala D, Bienkowski M, Stoczynska-Fidelus E, Gresner SM, Piaskowski S, Jaskolski DJ, Papierz W, Zakrzewski K, Kolasa M, Ironside JW, Liberski PP (2010) Glioblastoma-derived spheroid cultures as an experimental model for analysis of EGFR anomalies. J Neurooncol 2010 (E-pub ahead of print 29 August 2010; doi:10.1007/s11060-010-0352-0) 10.1007/s11060-010-0352-0 PMC3089721 20803305

[bib9] Zhao S, Lin Y, Xu W, Jiang W, Zha Z, Wang P, Yu W, Li Z, Gong L, Peng Y, Ding J, Lei Q, Guan KL, Xiong Y (2009) Glioma-derived mutations in IDH1 dominantly inhibit IDH1 catalytic activity and induce HIF-1alpha. Science 324: 261–265 19359588 10.1126/science.1170944 PMC3251015

